# Exploring links between personality traits and their social and non-social environments in wild poison frogs

**DOI:** 10.1007/s00265-022-03202-9

**Published:** 2022-07-04

**Authors:** Mélissa Peignier, Yimen G. Araya-Ajoy, Lauriane Bégué, Sarah Chaloupka, Katharina Dellefont, Christoph Leeb, Patrick Walsh, Max Ringler, Eva Ringler

**Affiliations:** 1 Division of Behavioural Ecology, Institute of Ecology and Evolution, University of Bern, Wohlenstrasse 50a, CH-3032 Hinterkappelen, Bern, Switzerland; 2 Messerli Research Institute, University of Veterinary Medicine Vienna, Vienna, Austria; 3 Department of Behavioral and Cognitive Biology, University of Vienna, Vienna, Austria; 4 Department of Biology, Centre for Biodiversity Dynamics, Norwegian University of Science and Technology, Trondheim, Norway; 5 Department of Biology and Ecology, University of Montpellier, Montpellier, France; 6 Department of Evolutionary Biology, University of Vienna, Vienna, Austria; 7 iES Landau, Institute for Environmental Sciences, University of Koblenz-Landau, Mainz, Germany; 8 Central Research Laboratories, Natural History Museum Vienna, Vienna, Austria; 9 Institute of Ecology and Evolution, School of Biological Sciences, University of Edinburgh, Edinburgh, UK; 10 University of Music and Performing Arts Graz, Graz, Austria

**Keywords:** Behavioral variation, Animal personality, Poison frogs, Non-random distribution, Environment

## Abstract

An animal’s behavioral phenotype comprises several traits, which are hierarchically structured in functional units. This is manifested in measured behaviors often being correlated, partly reflecting the need of a coordinated functional response. Unfortunately, we still have limited understanding whether consistent differences in animal behaviors are due to underlying physiological constraints or a result of plastic adaptation to their current environment. Therefore, characterizing the spatial distribution of behaviors can provide important insights into causes and consequences of behavioral variation. In the present study, we quantified behaviors in a wild, free-ranging population of the Neotropical frog *Allobates femoralis*. We investigated how these behaviors were linked to the frogs’ natural and social environment and quantified the extent to which these behaviors consistently differed among individuals (i.e., animal personality). We assessed levels of aggressiveness, exploration, and boldness by measuring several underlying behaviors expressed in a set of experimental assays, and found evidence for consistent among-individual differences along these axes. Contrary to our expectation, there was no relationship between individual behaviors and their natural environment, but we found a plastic response of males to changes in female density, which might reflect how individuals cope with their socio-ecological environment.

**Significance statement:**

How are behavioral phenotypes distributed across space? Here, we studied an entire free-ranging population of poison frogs, and investigated if the personality traits aggressiveness, exploration, and boldness are linked to the frogs’ natural or social environment. We found that behavioral traits were non-randomly distributed across the population, suggesting that the spatial arrangement of behavioral traits reflects how individuals cope with their complex natural and social environment.

## Introduction

Behavioral variation is ubiquitous in nature. Behaviors may vary considerably among species; and within species, we find variation both among and within individuals. Behavioral variation at all of these levels plays a major role in reproduction and survival, for example, affecting the risk of being detected and caught by predators, likelihood of dispersal, foraging efficiency, and/or the attractiveness to mating partners (Pigliucci 2005; Réale and Dingemanse 2010; Sih et al. 2012). This variation can occur in the form of consistent among-individual differences in behavior, referred to as animal personality, which has been documented in many animal taxa (Bell 2005; Dochtermann and Jenkins 2007; Tremmel and Müller 2013; Zidar et al. 2017; Goursot et al. 2019). Animal personality, however, does not preclude the existence of individual plasticity (Dingemanse et al. 2010), and we need to take into consideration both sources of variation when thinking about adaptive significance of behavioral variation in animals.

Five main personality traits are generally characterized in animals along the following axes: active/passive, aggressive/docile, bold/shy, exploratory/stationary, and sociable/non-sociable (Réale et al. 2007). Personality traits can be seen as latent variables that affect multiple quantifiable behaviors of an organism in certain contexts (Pigliucci 2003; Araya-Ajoy and Dingemanse 2014). For instance, aggressiveness can be seen as an unobservable (i.e., latent) variable that affects several observable behaviors during agonistic encounters, which can be assessed and quantified in an experimental context (e.g., speed of territorial reaction, number of attacks). Situations where personality traits are non-randomly distributed across space are typically referred to as “phenotype by environment correlation,” where environment refers to both the natural and social surroundings of the individual (Conover and Schultz 1995; Dingemanse and Araya-Ajoy 2015). For example, anemones (*Condylactis gigantea*) living in areas with higher seagrass were found to be shyer than those living in more open areas (Hensley et al. 2012) and western bluebirds (*Sialia mexicana*) modify their aggression to match the level of aggressive behavior of their mate (Duckworth and Kruuk 2009).

Since personality traits can vary simultaneously within and among individuals, associations between behavior and environment can originate from multiple processes, such as a non-random distribution of behaviors and/or phenotypic plasticity (Sprau and Dingemanse 2017). A non-random distribution of behaviors might be a direct effect of certain phenotypes (caused by genes and permanent environmental effects) showing a preference for certain environments. Alternatively, selective pressures induced by the heterogeneity of the environment can also maintain or generate individual differences in behavior within a population (Dingemanse et al. 2004). For instance, in territorial species, only highly aggressive individuals may succeed in establishing a territory in high-density patches, where they have access to more mates but face elevated intra-sexual competition. In turn, the link between aggressiveness and population density could also be caused by individual plasticity allowing individuals to match their aggressiveness to competition levels. Regardless of the mechanisms linking behavior and environment, identifying non-random spatial distribution of behaviors provides important insights into their function and their role in allowing organisms to cope with environmental variation. Long-term studies of the distribution of behaviors in the wild are thus a necessary first step towards understanding the mechanisms underlying the non-random distribution of behavioral traits (Archard and Braithwaite 2010).

Amphibians, and in particular Neotropical poison frogs (Dendrobatidae, sensu AmphibiaWeb 2022), are great models to study behavioral variation across their environment. Many species show territoriality or site fidelity that facilitates repeated measurements in wild individuals (Wells 2007; Kelleher et al. 2018), which can then be linked to local environmental parameters. Many species also exhibit elaborate courtship behavior, terrestrial oviposition, and obligatory tadpole transport of hatched larvae to aquatic sites (e.g., Crump 1972; Roithmair 1994; Pröhl 2005; Pašukonis et al. 2013; Rojas and Pašukonis 2019; Yang et al. 2019; Souza et al. 2021), offering ideal prerequisites for within- and between-individual variation in behavior to arise. In the present study, we aim to quantify how male-male aggression, exploratory, and anti-predator behaviors are expressed as functional units that can be described as latent variables reflecting three personality axes (referred to by one dimension of the axis as “aggression,” “exploration,” and “boldness”). We also aim at identifying how behaviors relate to the individual’s natural and social environment using a free-ranging population of the Neotropical poison frog *Allobates femoralis* (Dendrobatidae).

Based on the natural history of *A. femoralis*, we make multiple predictions. In males, we expect to find consistent among-individual differences in boldness and aggression. During the reproductive season, males produce loud advertisement calls to warn intruders and to attract females (Ringler et al. 2011; Chaloupka et al. 2022). Differences in personality traits across males might be related to differences in the trade-off they face between calling to secure mating and exposure to predators. As females do not display territoriality but merely perch fidelity (Fischer et al. 2020) and are never observed in any other aggressive interaction, they are generally considered non-aggressive; we have no clear expectation if and how varying degrees of boldness could be maintained in females. However, we expect to find consistent individual differences in exploration in both sexes, because males transport tadpoles to natural pools located up to 200 m away from their territory and females commute to male territories within 20 m distance of their perch for mating (Ringler et al. 2012, 2013, 2018; Fischer et al. 2020). Finally, we expect males’ personality traits to be non-randomly distributed across space because individual males face distinct challenges based on their social and/or natural environment. On the one hand, we expect more aggressive and bolder individuals to occupy areas of low complexity (e.g., with sparse vegetation and few ground structures) where they are easier to spot for females, while more passive or shyer individuals should occupy areas of higher complexity with more places to hide. On the other hand, we expect bolder and more aggressive individuals to occupy territories in areas with a higher population density than those occupied by more passive or shyer individuals.

## Methods

### Study population and area

We conducted our study in a population of *A. femoralis* on a lowland rainforest river island of approximately 5 ha. The island is situated in the “Les Nouragues” nature reserve in French Guiana (4°02′ N, 52°41′W; Bongers et al. 2001), near the “Saut Pararé” field camp of the CNRS Nouragues Ecological Research Station. The population was introduced in 2012 and has reached a stable size of approximately 150 adult individuals (see Ringler et al. 2014). The experiments took place from February to April of 2019, coinciding with the breeding season. We surveyed the population every day during its most active hours from 0900 to 1800 h, aiming to sample all adult males and females on the island in the course of the study period.

We caught frogs using a transparent plastic bag to minimize stress and direct contact, thereby limiting the potential influence of handling on behaviors. We identified all frogs via digital pictures of their distinct ventral patterns and with the help of the pattern matching software Wild-ID (Bolger et al. 2012), and sexed them by the presence (males) or absence (females) of vocal sacs. Ventral patterns clearly differ among individuals and are consistent across their adult lifespan, providing a reliable method of identification. Information on the age of individuals was available from a concurrent longterm monitoring project of the island’s population since its origins in 2012. We recorded the locations of all frogs on a detailed digital map (Ringler et al. 2016) using the mobile GIS software ArcPad 10 (ESRI, Redlands, CA, USA) on rugged Win10 tablets (CAT T20, Bullitt Group, Reading, UK), and further handled the data in ArcGIS 10.6 (ESRI). We determined body size of all adults (snout urostyle length; SUL) from dorsal photographs in front of a reference scale using the software Image J 1.52a (Rasband 1997–2021).

### Quantification of aggressive behaviors

**Fig. 1:**
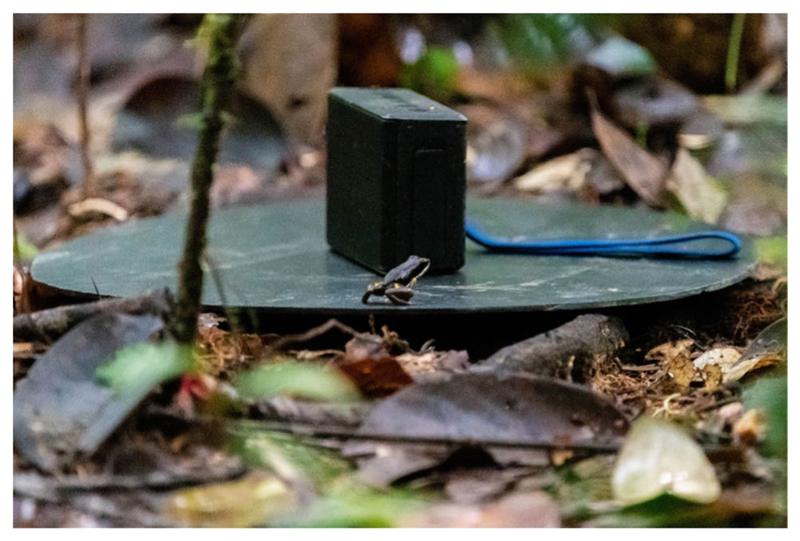
Picture of the territorial defense trial, presenting the speaker and a male on top of the perimeter

Aggression is often measured via “an individual’s agonistic reaction towards a conspecific” (Réale et al. 2007). To assess individual levels of aggression, we used acoustic playbacks to evoke territorial defense behavior in focal males (Fig. 1; Ursprung et al. 2009). During agonistic encounters, male *A. femoralis* display typical responses consisting of an orientation of their head/body, jumps towards the intruder, and sometimes direct attack (i.e., wrestling) (Hödl 1987; Narins et al. 2003). We expect that in the context of territorial defense, the personality trait “aggression” affects the latency until the first head-body orientation, the latency to the first jump, the probability to jump during moments when the intruder does not call, and the speed to approach the intruder (Chaloupka et al. 2022). Aggressive territorial behavior was only assessed in males, as females do not defend territories.

We used a music player with integrated loudspeaker (MUVO 2c, Creative, Singapore), centered on top of a black PVC disc (radius = 15 cm), 2 m from and facing the calling focal male (Fig. 1). We established the 2 m distance between the focal frog and the loudspeaker with a laser rangefinder (DLE 50, Bosch, Stuttgart, Germany). The experimenter remained a further 1 m behind the loudspeaker. We gave a 30-s acclimation period to the frog, which was enough for the frog to return to normal behavior and calling, before randomly presenting one of seven synthetic calls. The calls varied in their inter-note and inter-call intervals to avoid habituation over the course of the experiment. Each synthetic call featured the spectral and temporal parameters of a nearby free-ranging population of *A. femoralis* (Narins et al. 2003; Gasser et al. 2009). Each of the playbacks lasted for 5 min and was presented from the original WAV-files (16-bit, 44.1 kHz) using the same volume settings across all trials.

Using a digital voice recorder (ICD-PX333, Sony, Tokyo, Japan), we commented on the behavior of the focal male, noting its first head-body orientation, its jumps, and its arrival at the speaker (i.e., touching the disc with at least one part of his body). The trial ended when the male entered the 15-cm perimeter or when the playback stopped after 5 min. Trials where a focal male began to call were stopped and excluded from further analysis, as this was most likely the result of the speaker being positioned outside the defended area of the focal male’s territory (cf. Ringler et al. 2011). Vegetation density may cause the sound to be reflected and/or distorted, so we measured the received sound pressure level (“SPL” in dB) of the signal after each trial using a sound pressure meter (SL-100, Voltcraft, Hirschau, Germany). In all cases, the received SPL was above the threshold of 56 dB to elicit a behavioral response (Hödl 1983). The minimum duration between two consecutive tests with the same individual was 24 h (on average tests were 12.11 ± 7.92 SD days apart). *Allobates femoralis* typically lives for 1.5 years (Ringler et al. 2009; Ursprung et al. 2011a, b) and males only display aggressive reactions to territory intruders during the reproductive season. In our study, we aimed to test individuals with similar among- and within-individual inter-test intervals to cover several weeks of the reproductive season. Therefore, an average interval of 12 days between tests represents an effective compromise across the available time and life span of the species.

We blinded audio file names prior to analysis and coding to avoid observer bias. From the dictaphone recordings of the territorial defense trials, we extracted the latency until the first head-body orientation (in s), the latency until the first jump (in s), the speed to reach the speaker (in cm/s), and if the male jumped (1) or not (0) during inter-bout-intervals (hereafter “inter-bout jumps,” cf. Ursprung et al. 2009) using the software VLC (VideoLAN 2021). Males that did not reach the speaker were coded a censored speed of 0 cm/s. Following the same reasoning, males that did not perform a head-body orientation or a jump were given a censored latency of 300 s (corresponding to the total duration of the experiment). In total, we conducted 163 valid territorial defense trials with 51 males (mean ± SD = 3.20 ± 1.31 repetitions per individual).

### Quantification of exploratory tendency and boldness

**Fig. 2:**
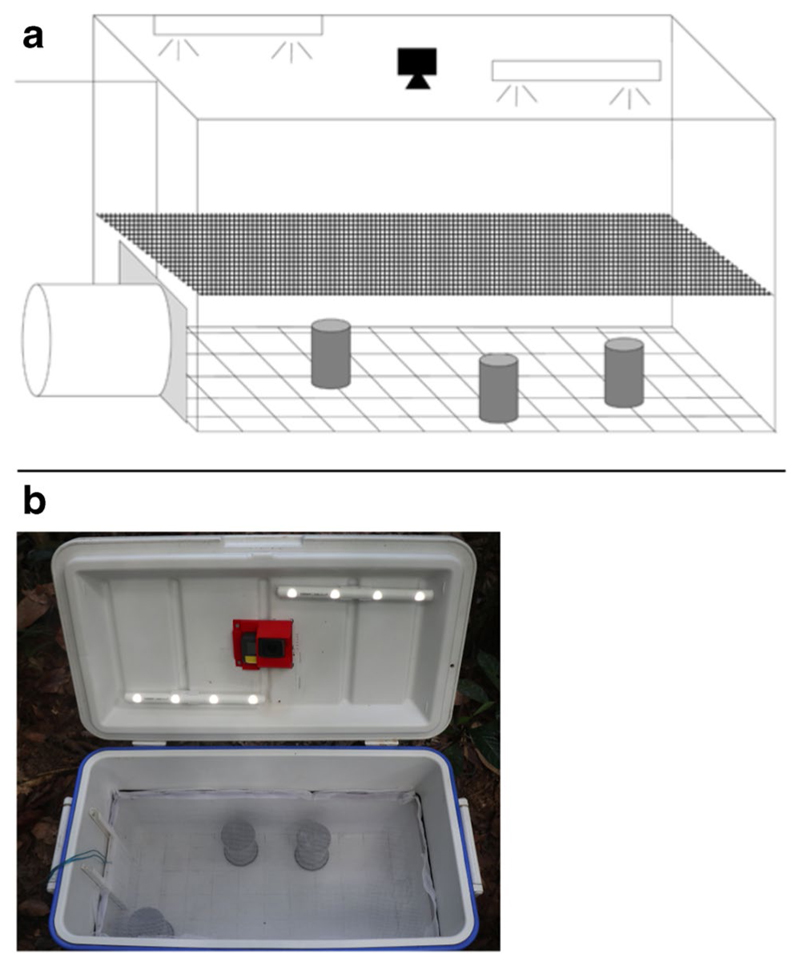
Schematic (**a**) and picture (**b**) of the Novel Environment Test. The cooler box measured 50 × 25 × 29 cm, with a 10 cm PVC tube attached on one side of the box. A sliding door separated the shelter from the box. Two LED tubes and a Hero Black 5 Go pro camera were attached to the lid of the box. Three solid PVC tubes (10 cm height, 5 cm diameter) were placed inside the box as visual obstacles at randomized positions every day. A grid was drawn on the floor of the cooler box to help randomize the position of the obstacles. A mesh net was placed in the cooler box at 20 cm height to prevent the frog from jumping on a wall outside of the camera range

To assess levels of exploratory tendency and boldness in male and female *A. femoralis*, we used a Novel Environment Test (NET), an approach that has been commonly used in personality studies (Carter et al. 2013; Kelleher et al. 2018). “Boldness” (or the corollary “shyness”) is defined as “an individual’s reaction to any risky situation,” and “exploration” as “an individual’s reaction to a new situation” (Réale et al. 2007). Therefore, we expected the personality trait “boldness” to affect the latency to go out of a dark shelter into a bright (novel) environment and the probability to enter the novel environment, and the personality trait “exploration” to affect the distance travelled, the number of jumps performed, and the area covered in the novel environment. Those behaviors were chosen in accordance with previous amphibian personality studies (Kelleher et al. 2018). Although counterintuitive at first, the number of jumps and the area covered are both important aspects of a frog’s exploratory tendency. Indeed, two individuals performing an equal number of jumps can visit more or fewer distinct areas of the box depending on their respective average jump lengths. Individuals were tested at the same location they were found. Males were tested immediately after the territorial defense test, and females were caught at their encounter location and put straight inside the NET (Fig. 2). Although this method prevents us from comparing levels of exploratory tendency and boldness between sexes, it was chosen to keep handling as minimal as possible on males.

The NET setup consisted of a cooler box (50 × 25 × 29 cm, hereafter “Novel Environment”), with a 10-cm PVC tube attached on one side of the box (hereafter “shelter”). An opaque sliding door separated the Novel Environment from the shelter. In the lid of the box, we installed a wide-angle video camera (Hero Black 5, GoPro, San Mateo, CA, USA) and two elongated, battery powered LED lights (LUMIstixx, Osram/Ledvance, Garching, Germany) for homogeneous illumination. We set the camera to “superview” mode to ensure full visibility of the Novel Environment. We installed a mesh net in the Novel Environment at 20 cm height to prevent the frog from jumping on a wall outside of the camera range. We used three solid PVC tubes (10 cm height, 5 cm diameter) as visual obstacles, to motivate the frogs to explore the entire Novel Environment. The positions of the obstacles were changed daily and their positions were determined using a random number generator.

At the beginning of each trial, we placed all individuals in the shelter for 10 min to allow them to acclimatize. The shelter remained accessible throughout the trials to encourage natural behaviors within the Novel Environment (Carter et al. 2013; Kelleher et al. 2018), as the individual was free to remain inside or return to the shelter at any time. After this acclimation period, we switched on the lights and the camera, closed the lid of the Novel Environment, opened the sliding door between shelter and Novel Environment, and filmed for 15 min. The minimum duration between two consecutive tests with the same individual was 24 h (on average tests were 11.36 ± 8.43 SD days apart).

We blinded video file names prior to analysis and coding to avoid observer bias. To analyze the video recordings obtained during the NET, we used the software TRACKER (Brown 2019) to correct for distances that were distorted by the camera wide-angle lens. Using the coding software BORIS (Friard et al. 2016), we assessed the latency from the opening of the sliding door until frogs left the shelter (in s) and the number of jumps performed inside the Novel Environment. For individuals who stayed inside the shelter during the entire experiment, the time spent in the shelter was censored to a value of 900 s (corresponding to the total duration of the experiment). We also coded the decision to enter the Novel Environment (1) or not (0) as a binomial response. Using the automated tracking software TOXTRAC (Rodriguez et al. 2018), we measured the distance travelled (in pixels) as well as the area visited inside the Novel Environment. For the latter variable, the software divided the floor of the Novel Environment into a 9 × 8 grid and automatically counted the number of distinct squares a frog visited during a trial. As individuals varied in the time they spent inside the Novel Environment, we standardized the data collected by only considering movements performed during the first 2 min after leaving the shelter. On average, individuals spent 624.21 s inside the Novel Environment (± 303.13 SD). This timeframe allowed for meaningful levels of movement to occur but maximized the available data. Using this criterion, 21 out of 259 tests had to be excluded from the exploration analysis, leaving a total of 238 valid NET trials with 52 males and 35 females (mean ± SD = 2.74 ± 1.33 repetitions per individual). Only very few individuals did not leave the shelter in all repetitions.

### Territory size, habitat complexity, and number of neighbors

To find out the distribution patterns of behaviors with respect to a male’s natural and social environment, we determined the territory size and the local complexity of the habitat, as well as the number of female and male neighbors for each male. This was only studied in males, as in *A. femoralis* males acquire and defend non-overlapping territories, while female display only site fidelity (Ringler et al. 2009, 2011 2012; Fischer et al. 2020). We used Dirichlet tessellation in ArcGIS to approximate male territories as Voronoi polygons (Voronoi 1908) on a day-to-day basis (for more information, see Supplementary Materials). For the daily territory estimation, we applied a roving-window approach, using all capture points of the last 5 days a male was seen, including the focal day, but excluding all points where a male was likely found outside its territory (i.e., all capture points linked to tadpole transport). To ensure that the sizes of territories located at the population periphery were not over-estimated (Ringler et al. 2009), we included the vertices of the island outline into the Dirichlet tessellation procedure, to establish a buffer from the edge of the island until halfway to the outermost capture points of peripheral males. To ensure that estimates at the beginning and end of the season were not impacted by individuals not being captured, we used the POPAN formula (Schwarz and Arnason 1996) in program MARK (White and Burnham 1999; White 2020) to correct our population estimates at these points in the breeding season (for more detailed information, see Supplementary Materials).

After conducting the Dirichlet tessellation, we dissolved the resulting Voronoi polygons based on male identity to obtain “Voronoi territories” for each male and then calculated territory sizes in ArcGIS. We also counted the direct male neighbors for each focal male from the daily Voronoi territories. To obtain the number of female neighbors, we calculated female centroid points (mean center) across the entire season. Based on the typical distances females commute for mating (Ringler et al. 2012; Fischer et al. 2020), we constructed 20-m buffer circles and counted the number of contained centroid points of male Voronoi territories.

To determine the complexity of the habitat for each individual male, we took four photographs (camera in automatic (P) mode, focal length 50 mm, no flash, jpg images) from each of the cardinal directions (0°, 90°, 180°, and 270° towards magnetic north) of a 100 cm × 100 cm red fabric. The fabric was placed at 3.5 m from the centroid point of each male’s territory at forest floor level as this represents half the radius of the typical male territory (cf. Ringler et al. 2011). We positioned the camera 20 cm above the forest floor, corresponding to the perch location of a male during territorial advertisement calling. We calculated habitat complexity as the average percentage of red fabric that was covered by vegetation on the four pictures, with increasing coverage of the fabric representing more complex habitat. For that, we cropped each image in Paint.net v4.1.6 (Brewster 2019) to show only the lower half of the red fabric as this would represent the most visually relevant part of the habitat for the frogs. Finally, we counted the number of pixels of visible fabric to calculate the percentage of fabric covered by vegetation. To facilitate this process, we aimed at increasing the differences between fabric and vegetation by setting the image hue to 180 and decreasing the luminance and contrast to − 20. If males changed their territory location during the study period, analysis of habitat complexity was performed in the territory in which most experimental trials had been conducted.

## Statistical analysis

We conducted all statistical analyses in R v3.6.0 (R Core Team 2020) using the integrated development environment RStudio v1.3.1093 (RStudio Team 2019).

## Structure of behaviors

To determine how the measured behaviors are structured into functional units (i.e., aggression, exploration and boldness), we investigated the phenotypic covariance structure among different measurements during the behavioral tests. To infer a latent variable describing aggression from behaviors measured during the territorial defense test (e.g., speed to reach the speaker, latency until the first head-body orientation, latency until the first jump, inter-bout jumps), using the SEM package, we applied structural equation modelling to the phenotypic covariance matrix derived from the means of each behavior for each individual, in order to avoid pseudo-replication (Fox et al. 2020). Models were compared, to determine the most parsimonious model (for details see Supplementary Fig. 1), based on differences in Akaike’s information criterion (AIC) values, with small values indicating higher parsimony and a ΔAIC ≥ 2 indicating significant differences (Burnham and Anderson 2002). We expected the best model to have a latent variable explaining the covariance among the four measurements. We applied the same technique to determine whether two latent variables describing boldness and exploration can be inferred from the behaviors measured during the NET (i.e., distance travelled, number of jumps, number of areas, time spent in the shelter, and probability to enter the box; for details see, Supplementary Fig. 2). We expected the best model to have two correlated latent variables, one that would explain explorative behaviors (e.g., distance travelled, number of jumps, number of areas) and one that would explain boldness related behaviors (e.g., probability to enter the box, time spent in the shelter). In addition, Bayesian generalized linear mixed models were used to confirm that there were no impacts from the fact that SEM analysis combines within and between individual effects (for details see Supplementary Material).

### Personality traits

To investigate if *A. femoralis* exhibits personality (i.e., between-individual variation in behaviors), we assessed the repeatability (R) of all measured behaviors using the “rpt” function in the rptR package (Stoffel et al. 2017). Latency until the first head-body orientation and until the first jump had to be log transformed to achieve normal distribution. We used the function “transformTukey” to apply a constant transformation on the speed to reach the speaker, the distance travelled, and the time spent in the shelter. Repeatability was estimated based on the models applied to the transformed data. We estimated repeatability from models fitted with a Gaussian error distribution for the latency until the first head-body orientation and until the first jump, the speed to reach the speaker, the time spent in the shelter, and the distance travelled. We estimated repeatability from models fitted with a Poisson error distribution for the number of jumps and the number of areas, and from models fitted with a binary distribution for the inter-bout jumps and the probability to enter the box. For all models, ID was included as a random effect.

### Distribution of behaviors across the natural and social environment

**Fig. 3:**
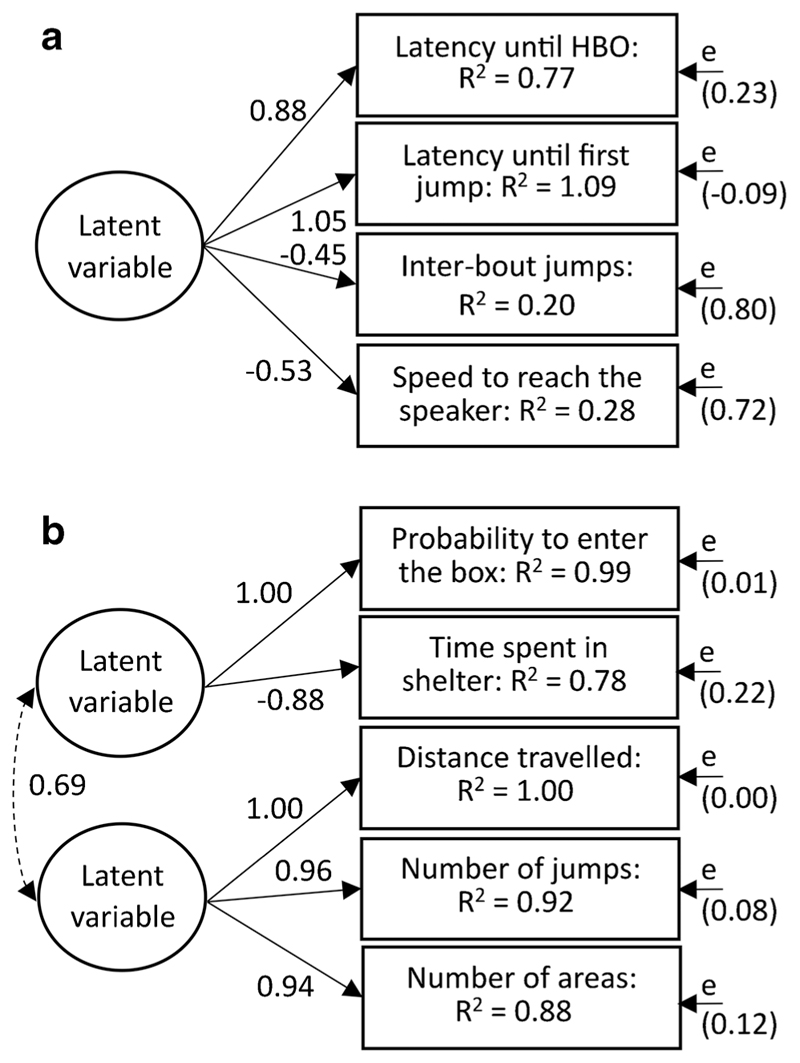
Path diagrams of the best structure equation models (SEMs) (based on difference in Akaike’s information criterion (AIC) values) explaining the covariance structure among four behaviors assessed during an aggressivity test (**a**), and five behaviors assessed during a Novel Environment Test (**b**). “HBO” refers to the latency until the first head-body orientation. Squares represent the variances of the different behaviors explained by the SEM structure (R^2^). Numbers associated with arrows are standardized factor loadings which represent how behavioral responses are predicted to change based on changes to the latent variable. Number in brackets represent variances of residuals or error variances (e) associated to each behavior. All simulated models can be found in the Supplementary material (Supplementary Fig. 1, 2)

We used a bivariate approach to study how behaviors measured during the territorial defense trial and the NET correlate with variation in the habitat complexity, territory size, and number of male and female neighbors at the among- and within-individuals level using Bayesian generalized linear mixed models (Hadfield 2010). In the analyses, the latency until the first jump, distance travelled, and time spent in the shelter were chosen as they best represented the latent variables of aggression, exploration, and boldness, respectively (see the “Structure of behaviors” section and Fig. 3). Although the probability to go inside the box was slightly more correlated to boldness than “time spent in shelter,” we decided to use the latter variable to avoid using a binomial variable in the model. Models were built with the transformed data (see the “Personality traits” section).

To investigate the among-individual covariance between behaviors and environment variables, we divided each of the environmental variables by their mean value and added all of them as response variables (see Houslay and Wilson 2017).

We included age (i.e., as a binomial trait: newly encountered adults vs. recaptures from previous years) as a traitspecific fixed effects to control for effects of age on all our response variables. To calculate the variance due to differences among individuals and the covariance between measured behaviors, we fitted an unstructured covariance matrix for the grouping variable ID. We then used the posterior distributions to estimate the among- and within-individual correlations and covariances between each of the behavior measured and the environment. We assumed statistical significance if the 95% credible intervals did not overlap 0 and performed the same model verification as previously (see the “Structure of behaviors“ section). We also fitted these relationships between behaviors and environment variables with univariate linear mixed models corrected for multiple comparisons, which led to the same biological conclusions.

## Results

### Personality traits

**Fig. 4:**
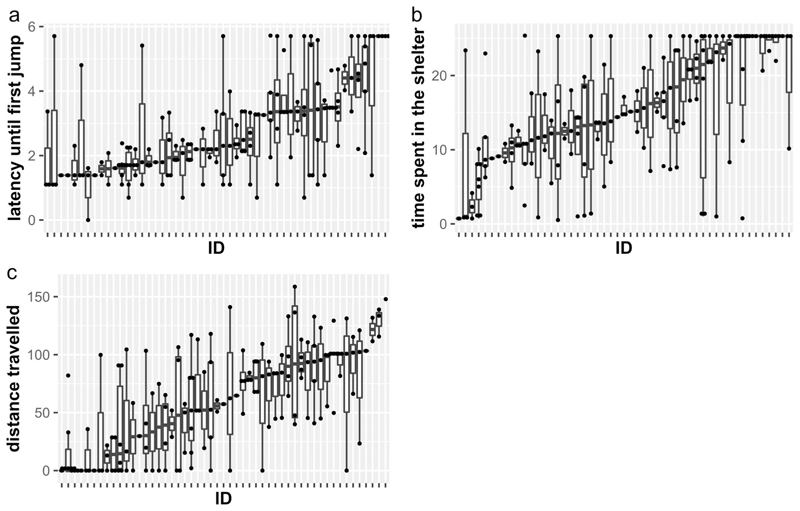
Range of variation in the three behaviors that best represented the latent variables of aggression, exploration and boldness. The latency until the first jump (**a**), the time spent in the shelter (**b**), and the distance travelled in the Novel Environment Test (**c**) are presented for individual males. All variables have been transformed using a log (**a**) or constant (**b** and **c**) transformation. Boxes indicate the inter quartile range, with the central line depicting the median and the whiskers extending to 1.5*IQR. Dots represent the results of each trial. Males are ordered by their median (represented as a horizontal bold line)

Among the measures taken from the territorial defense test, all were considered repeatable and ranged in repeatability from 0.17 to 0.37 (Table 1; Fig. 4a). The best SEM model supports a latent variable explaining the covariance of the four behavioral responses measured in a territorial defense test (Fig. 3a; Supplementary Fig. 1b). Together, these results suggest that *A. femoralis* exhibits a personality trait “aggressiveness” encompassing the latency until the first head-body orientation and until the first jump towards a calling intruder, the speed to reach the intruder, and the probability to jump during inter-bout. However, the residual variances were high (Fig. 3a; Supplementary Fig. 1b) and another model encompassing two latent variables had an AIC score close to the best model (ΔAIC = 1.7; Supplementary Fig. 1 g).

Similarly, we measured individual behavioral responses in a NET, where we found significant repeatabilities in all behavioral measurements (Table 1; Fig. 4b, c). Based on the comparison of the SEM, the best model supported the existence of a latent variable explaining the covariance patterns of three behavioral measurements (distance travelled, number of jumps, and number of areas). It also supports that another latent variable can be derived from the covariance of two behavioral measurements (time spent inside the shelter and probability to enter the box). The two latent variables are correlated (Fig. 3b; Supplementary Fig. 2 h). Together, these results suggest that *A. femoralis* males and females exhibit a personality trait “exploration” encompassing the distance travelled, the number of jumps performed, and the area covered in a new environment. The results also suggest the existence of the personality trait “boldness,” encompassing the latency to leave a safe place and enter a new environment and the probability to enter a new environment.

For both the behaviors measured in the territorial defense test and in the NET, the phenotypic covariances in the SEM were mostly driven by the within-individual variances. The results of the Bayesian models also show that the direction of the among- and within-individual covariances was similar (Supplementary Tables 2, 3). Therefore, doing the SEM using only the among- or within-individual covariance matrix would have resulted in a similar interpretation of the results.

### Behavior and the characteristics of an individual and its environment

Next, we investigated the correlation between the behaviors measured during the territorial defense trial and habitat complexity, territory size, and the number of male and female neighbors. On average, habitats were relatively complex with 82.77% of the fabric covered by vegetation in the measure of habitat complexity (SD = 9.31). Males occupied territories of 669.31 m^2^ on average (SD = 440.99). Males had on average 7 female and 5 male neighbors (mean of individual means) across the season (absolute range females: 1–15, absolute range males: 1–10). Throughout the season, the average variation in the number of both female and male neighbors was 2 for individual territory holders (range females: 0–7; range males: 0–6).

**Fig. 5:**
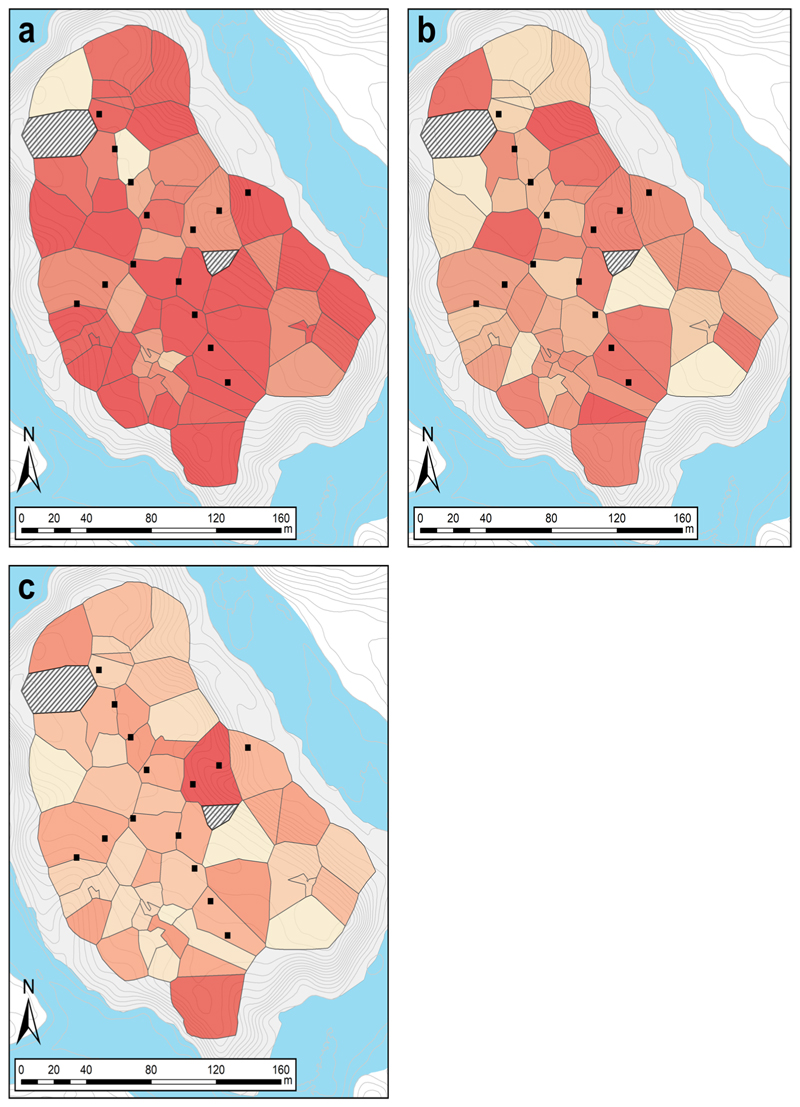
Maps showing the spatial distribution of individual performance of male frogs on the island in the behavioral essays. The maps use the Voronoi territories of 7 March 2019 when the most individuals were present at the same time on the island. The maps show the mean value, calculated over all respective trials of (**a**) the individuals’ latency to jump in the territorial defense trial, (**b**) the time spent in the shelter during the Novel Environment Test (NET), and (**c**) the distance travelled in the NET. All color ramps have 20 equal intervals across the full range of the respective value; darker colors represent shorter latency in (**a**), shorter time spent in the shelter in (**b**), and longer distances in (**c**). Black squares indicate the 14 artificial pools that were in place on the island since 2018; thin gray lines show 50 cm elevation isoclines; the blue area shows the river Arataye. The territories of two males that were not tested in the behavioral assays are shown with a hatched white pattern

There was no relation between the aggressive responses of a male during a territorial defense test and its social or natural environment. However, there was a significant relation between the level of exploration and boldness of a male and the number of female neighbors at the within-individual level (Supplementary Table 4). This suggests that individuals respond plastically to their social environment, increasing their level of exploration and boldness when the number of females around increases. The spatial setup of the territories on the day with the most individuals present at the same time (07th of March 2019) is presented in Fig. 5. The average response of each individual in terms of latency until the first jump, time spent in the shelter, and distance travelled in the box is also represented.

## Discussion

In the present study, we investigated the structure of potential personality traits in *A. femoralis* and determined how behaviors related to aggressiveness, exploration, and boldness are structured into functional units (i.e., personality traits). We also investigated how individual behaviors relate to an individual’s natural and social environment in a wild, free-ranging, entire population of *A. femoralis*.

### Personality traits

Our analysis showed that the repeatability of the variables measured in the territorial defense trial (0.17 to 0.37) was in the lower range of the repeatability found in most studies (with overlapping confidence intervals; Bell et al. 2009; mean = 0.37, 95% confidence limits = 0.35–0.38) but consistent with the average findings in other personality studies on amphibians (Brodin et al. 2013; Maes et al. 2013; Gifford et al. 2014; González-Bernal et al. 2014). Moreover, we found that a latent variable explains the covariance of the four behaviors measured during a territorial defense test (i.e., the latency until the first head-body orientation and until the first jump towards a calling intruder, the speed to reach the intruder and the probability to jump during the inter-bout interval). Together, these results suggest that *A. femoralis* males exhibit a personality trait “aggressiveness.” However, a great portion of the speed and the inter-bout jumps measured during the territorial defense trial were explained by exogenous factors. Together with the existence of another model with similar support, these results suggest that the behavioral measures capture different aspects of aggressiveness. In this model (Supplementary Fig. 1 g), a latent variable explains the covariance of the latencies until the first head-body orientation and until the first jump. Another latent variable explains the covariance between the speed to reach the speaker and the inter-bout jumps. We interpret these two latent variables as reactivity and offensiveness, respectively. Males of many amphibian species defend and fight over territories using acoustic and visual displays (Hödl and Amezquita 2001; Toledo et al. 2007). For instance, in the Bornean rock-skipper frog (*Staurois latopalmatus*), males perform foot-flagging and advertisement calls to defend their territories against conspecific intruders (Preininger et al. 2009). Our results show that more aggressive males react fiercer towards conspecific territory intruders. This is of particular ecological relevance, because territory possession is the most important prerequisite for male reproductive success in this species (Ursprung et al. 2011a). However, high levels of aggression might also come with a cost, if more aggressive individuals engage more often in energetically costly and potentially harmful fights.

We also found that the probability to enter a novel environment and the time it took to do so were highly repeatable, and a latent variable explained a relatively large part of the covariance among the two variables. Together, these results suggest the existence of the personality trait “boldness” in *A. femoralis* males and females. Boldness relates to the reaction of an individual to a predator, a novel object, or a conspecific and is relevant in many contexts such as predator avoidance, feeding, or mating (Réale et al. 2007). In *A. femoralis*, a generally high propensity to take risks might be reflected not only in the response to a predation threat, but also in how prominently the advertisement call is presented. Thus, higher risk taking could be reflected in males calling at higher rates, higher amplitude, over longer durations, from more exposed locations, or moving/turning more during calling. In *A. sub-folionidificans*, a closely related species, calling activity has been found to be positively related to reproductive success (Souza et al. 2021). Being bold, however, can also be costly as it might lead to more frequent encounters with predators. Future studies are needed to identify the link between calling activity and individual reproductive success, in order to investigate possible trade-offs in *A. femoralis*.

Finally, we found that the distance travelled, the number of jumps performed, and the area covered in a new environment were highly repeatable. Moreover, we found that a latent variable explained some of the covariance among the three behavioral measurements. Together, these results suggest that *A. femoralis* males and females exhibit a personality trait “exploration.” Exploration behavior is especially relevant for dispersal and resource acquisition (Dingemanse et al. 2003; Gruber et al. 2017). In *A. femoralis*, males rely on spatial memory to find water bodies (Pašukonis et al. 2016) and distribute their tadpoles across multiple sites to decrease risks of losing entire clutches due to desiccation or predation (Erich et al. 2015; Ringler et al. 2018). Therefore, being more explorative might enable males to find more sites for tadpole deposition or a better territory to settle, while females put their explorative behaviors at use when looking for a mate and, more rarely, for tadpole transport (Ringler et al. 2015). A highly explorative individual will, however, be more conspicuous to predators or at risk of losing its territory during periods of absence.

### Distribution of behaviors across the natural and social environment

Contrary to our expectation, we did not find a relationship between individual behaviors and their natural environment (i.e., habitat complexity and territory size). However, we cannot exclude that other habitat characteristics that we did not measure, such as quantity and quality of the leaf litter, tree species, or canopy cover, are linked to individual behavior. Likewise, we did not find any relationship between individual behavior and territory size, as more aggressive and bolder individuals settled in territories of varying sizes (Fig. 5a, b). This is in line with the previous finding that in *A. femoralis*, only the possession of a territory is important for reproductive success, but not territory size (Ursprung et al. 2011a), and as a consequence, males cannot increase their reproductive success by extending territorial space. As already suggested in a previous study, variation in territory sizes is probably strongly dependent on characteristics of the natural environment (Ringler et al. 2017) rather than a consequence of varying levels of aggression in *A. femoralis* males.

More explorative individuals were not necessarily located near the artificial pools on the island (Fig. 5c), although water bodies are of critical importance for individual fitness, given that they are obligate for tadpole development after hatching. Previous studies have shown that males distribute their tadpoles across multiple water bodies located outside their territories (Ringler et al. 2013, 2018; Erich et al. 2015; Beck et al. 2017). This suggests that the ability of an individual to find water bodies once it has settled in a territory is critical, while the location of the territory is not. Finding water bodies in *A. femoralis* is strongly related to olfactory sensing (Serrano-Rojas and Pašukonis 2021) and spatial memory might help in revisiting such sites once they have been encountered (Pašukonis et al. 2016; Beck et al. 2017). Taken together, our results suggest that *A. femoralis* males establish their territories independent of large-scale resource distribution and the wider structure of the habitat. And likewise, the characteristics of their natural environment apparently are not associated with individual differences in behavior.

We also did not find support for a relationship between aggressiveness and the social environment. We initially expected more aggressive individuals to occupy territories in high-density areas, where they would be more likely to find mating partners but also face elevated male-male competition. However, with this trade-off between female availability and intra-sexual competition, males might have equal reproductive outcomes regardless of their level of aggressiveness and independent of the overall density, as long as they manage to establish a territory at all. Future studies should investigate how the interplay between aggressiveness and population density affects reproductive success.

Our results show that exploration- and boldness-related behaviors were positively linked to the number of females in the vicinity to male territories. Since significant correlations were only found at the within-individual level, this suggests that the link between exploration or boldness and the social environment is mainly driven by individual plasticity. This indicates that males, who mostly have stable territories and move little, increase their overall levels of exploration and boldness when the number of females around their territory increases. There are currently no studies showing any direct mechanisms how males could assess the presence and number of nearby females. We suggest that future studies should investigate secondary or indirect cues, such as distribution or density of feeding sites, that might determine female density and that might allow males to identify areas with more females. However, because the among-individual effects have broad credible intervals that are not centered around zero, we cannot rule out the possibility of non-random settlement.

Our study does not claim or identify any causal relationship between behaviors or personality traits and their natural and/or social environment. Still, the identification of such relationship in a natural free-ranging population of animals provides a first step towards understanding the mechanisms underlying the distribution of behaviors across space (cf. Archard and Braithwaite 2010).

## Conclusion

We studied the structure of personality traits and the distribution of behaviors across the environment in an entire free-ranging population of the poison frog *A. femoralis* by measuring several behaviors in situ in different contexts. We found that *A. femoralis* indeed exhibits animal personality along the aggressiveness, exploration, and boldness axes. Furthermore, we found non-random distribution of behaviors across the animals’ social environment, which may allow individuals to cope with their complex socio-ecological environment. While amphibians have been largely overlooked in animal personality research, this study is one of the first comprehensive study of animal personality in amphibian in the wild. By providing a detailed description of how behavioral measurements are structured in functional units that allow individuals to cope with their socio-ecological environment, it broadens our understanding of the functional role of behavior in frogs and offers a first step towards understanding the mechanisms that play a role in the emergence and maintenance of behavioral variation.

## Figures and Tables

**Table 1:** Repeatability (R) and confidence intervals (CI) of the behaviors measured during the territorial defense test and the Novel Environment Test (NET)

Test	Variable	R	95% CI
Territorial defense test	Latency until head-body orientation	0.17	[0.02; 0.34]
	Latency until first jump	0.24	[0.07; 0.40]
	Speed to reach the perimeter	0.37	[0.19; 0.53]
	Inter-bout jumps	0.20	[0.001; 0.37]
NET	Probability to go in the box	0.44	[0.13; 0.89]
	Time spent in the shelter	0.30	[0.15; 0.44]
	Distance travelled in the box	0.36	[0.21; 0.49]
	Number of jumps	0.45	[0.27; 0.62]
	Number of areas visited	0.48	[0.30; 0.62]

## Data Availability

The datasets generated during and/or analyzed during the current study are available in the Open Science Framework repository: https://doi.org/10.17605/OSF.IO/AJV5X.
